# Statins and reporting signals for amnesia: a pharmacovigilance analysis based on adverse event reporting database

**DOI:** 10.3389/fphar.2026.1858243

**Published:** 2026-08-11

**Authors:** Le Wang, Nan Yang, Zhiyong Ren

**Affiliations:** 1 Shanxi Provincial Mental Health Center, Taiyuan Psychiatric Hospital, Taiyuan, China; 2 Shanxi Bethune Hospital, Shanxi Academy of Medical Sciences, Tongji Shanxi Hospital, Third Hospital of Shanxi Medical University, Taiyuan, China

**Keywords:** amnesia, atorvastatin, FAERS database, pharmacovigilance analysis, simvastatin, statins

## Abstract

**Background:**

This study aimed to explore the disproportionality reporting signals of amnesia associated with different statins based on a spontaneous adverse event database, so as to provide preliminary pharmacovigilance evidence for subsequent clinical safety evaluation and further pharmacoepidemiological verification.

**Materials and methods:**

This study employed pharmacovigilance analysis methods to monitor and analyze adverse event signals associated with seven commonly used statins. Additionally, subgroups were stratified by gender (male, female) and age (18, 45, and 65 years) to investigate the potential associations between statins and amnesia across different populations. Furthermore, a Weibull distribution analysis was used to assess the onset time of adverse events.

**Results:**

A total of 56,401 statin-related adverse event reports were included, among which 1,108 valid cases were coded as amnesia. Significant positive pharmacovigilance signals were identified for atorvastatin (n = 326, ROR = 2.82, 95% CI: 2.53–3.15, P < 0.001), simvastatin (n = 600, ROR = 4.94, 95% CI: 4.55–5.35, P < 0.001), pravastatin (n = 68, ROR = 4.77, 95% CI: 3.76–6.06, P < 0.001), and lovastatin (n = 26, ROR = 6.96, 95% CI: 4.73–10.24, P < 0.001). Subgroup analyses stratified by gender and age further identified heterogeneous pharmacovigilance signals across distinct populations. Moreover, Weibull distribution analysis also revealed unique distribution patterns among different statin agents.

**Conclusion:**

This study identified significant positive pharmacovigilance signals between certain statins and amnesia. The findings of this study provide preliminary disproportionality signals regarding statin-related amnesia, offering exploratory pharmacovigilance references for subsequent safety evaluation and further pharmacoepidemiological validation.

## 1 Introduction

Statins are a class of lipid-lowering drugs that target 3-hydroxy-3-methylglutaryl-coenzyme A (HMG-CoA) reductase (Thompson et al., 2016). By competitively inhibiting HMG-CoA reductase activity, they block a key step in cholesterol synthesis, thereby significantly reducing total cholesterol and low-density lipoprotein cholesterol levels in the body, while mildly increasing high-density lipoprotein cholesterol, exerting lipid-lowering and multifactorial protective effects (Ratchford and Martin, 2017). Commonly used statins are primarily classified into seven categories, including atorvastatin, simvastatin, pravastatin, lovastatin, fluvastatin, pitavastatin, and rosuvastatin (Cooper-DeHoff et al., 2022; Montastruc, 2023). Due to differences in metabolic pathways and potency, these drugs can meet the medication needs of various clinical scenarios (Sun et al., 2023; Yu et al., 2023). Currently, statins have become one of the most widely used lipid-lowering drugs globally, with extremely high prevalence, and are the first-line drugs of choice for the prevention and treatment of atherosclerotic diseases (Fan and Watanabe, 2022).

Amnesia is a group of neurocognitive disorders characterized primarily by impaired memory function (Cubelli and Della Sala, 2021; Langer, 2021). It is mainly manifested by a decline in the ability to recall past experiences, an inability to form new memories, or memory loss regarding specific time periods or events. Its onset is closely associated with structural or functional abnormalities in memory-related brain regions, such as the hippocampus (Markowitsch and Staniloiu, 2012). The severity of clinical symptoms varies; in severe cases, patients may completely lose the ability to care for themselves (Mayes, 1995). As a severe neurological disorder, amnesia not only leads to cognitive decline, preventing patients from working, studying, and socializing normally, but also causes them to lose awareness of their own identity and relationships with people.

As the clinical use of statins becomes increasingly widespread, and given that most patients require long-term or even lifelong treatment, related adverse events have gradually been widely reported and garnered significant attention (Jaam et al., 2023; Ko et al., 2019). In 2012, the U.S. Food and Drug Administration (FDA) issued a Drug Safety Communication and updated statin labeling. Besides, adverse events related to amnesia have gradually garnered widespread attention and reporting (Healy et al., 2009; Jamolowicz et al., 2015; Tan et al., 2019; Zhou et al., 2021). However, some studies have presented conflicting findings, suggesting that statins may actually improve the progression of this condition (Tapia-Perez et al., 2008; Yang et al., 2020; Westphal Filho et al., 2025).

The FDA Adverse Event Reporting System (FAERS), established and maintained by the U.S. Food and Drug Administration (FDA), is currently one of the most authoritative and influential large-scale post-marketing drug safety monitoring platforms in the global pharmacovigilance field. This database comprehensively aggregates information on adverse events related to FDA-approved drugs and biological products reported by healthcare professionals, consumers, and pharmaceutical manufacturers. It is updated quarterly, providing ample data resources for real-world studies on post-marketing drug safety (Yang et al., 2025). Since the potential association between statins and amnesia has not yet been clearly elucidated, and this association directly impacts patients’ long-term medication safety, this study aims to use the FAERS database as its data foundation and employ standardized pharmacovigilance analysis techniques to systematically identify potential disproportionality signals between seven commonly used statins and amnesia.

## 2 Materials and methods

### 2.1 Data sources

The U.S. Food and Drug Administration (FDA) Adverse Event Reporting System (FAERS) is a publicly accessible, post-marketing drug safety surveillance database that is updated quarterly; it is also one of the core data sources for pharmacovigilance research. Maintained and managed by the FDA, this database integrates adverse event information voluntarily reported by healthcare professionals, patients, and pharmaceutical companies, and is suitable for large-scale retrospective drug safety signal mining. This study included raw data from the FAERS database spanning the first quarter of 2005 through the first quarter of 2025. Specifically, the dataset comprises seven core modules: Demographic and Administrative Information (DEMO), Drug Information (DRUG), Adverse Event Coding (REAC), Patient Outcomes (OUTC), Reporting Source (RPSR), Treatment Duration (THER), and Indication (INDI). In accordance with official FDA specifications, CASEID, PRIMARYID, and drug_seq were used as unique identifiers for data linkage and sorting. For duplicate reports, the entry with the most recent FDA_DT (report submission date) was retained for the same PRIMARYID; for reports with matching CASEID and FDA_DT, the record with the higher PRIMARYID value was retained to reduce data redundancy and ensure stable and reliable analysis results.

### 2.2 Procedures

The Medical Dictionary for Regulatory Activities (MedDRA Version 26.1) was used in this study to standardize the coding of medical terms. This dictionary organizes clinical events into a four-level hierarchy comprising System Organ Classes (SOCs), Higher-Level Group Terms (HLGTs), Higher-Level Terms (HLTs), and Preferred Terms (PTs). To control for confounding factors, only reports in which the target statin was identified as the primary suspect drug (PS) were included, while records related to secondary suspect drugs, concomitant medications, and interacting drugs were excluded. A combined search using both generic and brand names was employed to comprehensively identify reports involving the target statins, and the raw data underwent rigorous cleaning to remove records with missing information, coding errors, and low-quality data. A classic 2 × 2 contingency table was used to perform a disproportionality analysis: cell a denotes the number of reports with exposure to the target statin and subsequent occurrence of amnesia; cell b denotes the number of reports with exposure to the target statin but without amnesia; cell c denotes the number of reports with exposure to other medications and occurrence of amnesia; and cell d denotes the number of reports with exposure to other medications but without amnesia (Supplementary Table S1).

### 2.3 Statistical analysis

This study employed a disproportionality analysis to detect signals of adverse events associated with statins and amnesia, incorporating four complementary algorithms: the Reporting Odds Ratio (ROR), the Proportional Reporting Ratio (PRR), the Information Component (IC), and the Empirical Bayesian Geometric Mean (EBGM). PRR and ROR are frequentist measures that compare observed reporting frequencies with expected values. IC and EBGM are Bayesian approaches that account for background reporting variability. Together, these methods enable robust and comprehensive detection of potential safety signals between statins and amnesia (Sakaeda et al., 2013). To reduce the risk of false positives, a safety signal was considered statistically significant only when it met the positive criteria of all four algorithms. Among these algorithms, the ROR demonstrates superior performance in mitigating bias and was therefore selected as the primary metric for comparing signal magnitudes after positive signal confirmation (Rothman et al., 2004). Additionally, Bonferroni correction was applied to adjust for multiple comparisons and control the Type I error rate. Specifically, this adjustment was performed based on the total number of PTs within each demographic subgroup for multiple testing. Furthermore, this study conducted subgroup analyses by gender (male, female) and age (≤18, 18–45, 45–65, >65 years) to explore differences in association patterns across different populations. Supplementarily, Weibull distribution analysis was employed to identify patterns in the timing of amnesia events and to reveal associated characteristics from a temporal perspective. The Weibull model is widely used in pharmacovigilance to describe time-to-event data, as it flexibly captures increasing, constant, or decreasing reporting rates over time. Its shape parameter reflects the trend of adverse event occurrence, while the scale parameter represents the typical time to event (Kinoshita et al., 2020). The shape parameter and scale parameter were estimated using the fitdistrplus package (version 1.2–6) in the R statistical environment. All data processing and statistical analyses were performed using R software (version 4.4.2).

## 3 Results

### 3.1 Descriptive analysis

**FIGURE 1:**
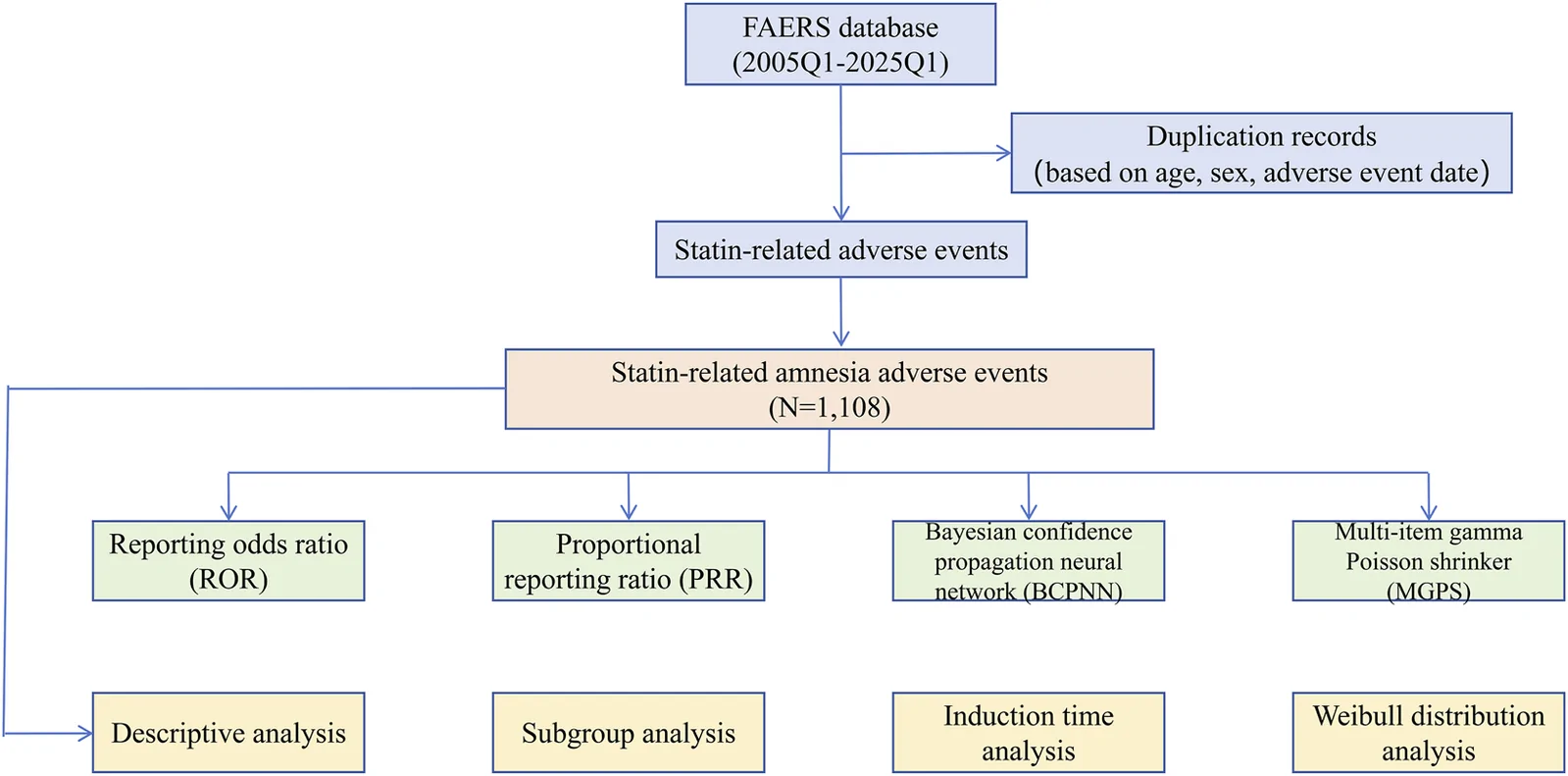
Flow chart showing the analysis process of the study.

**FIGURE 2:**
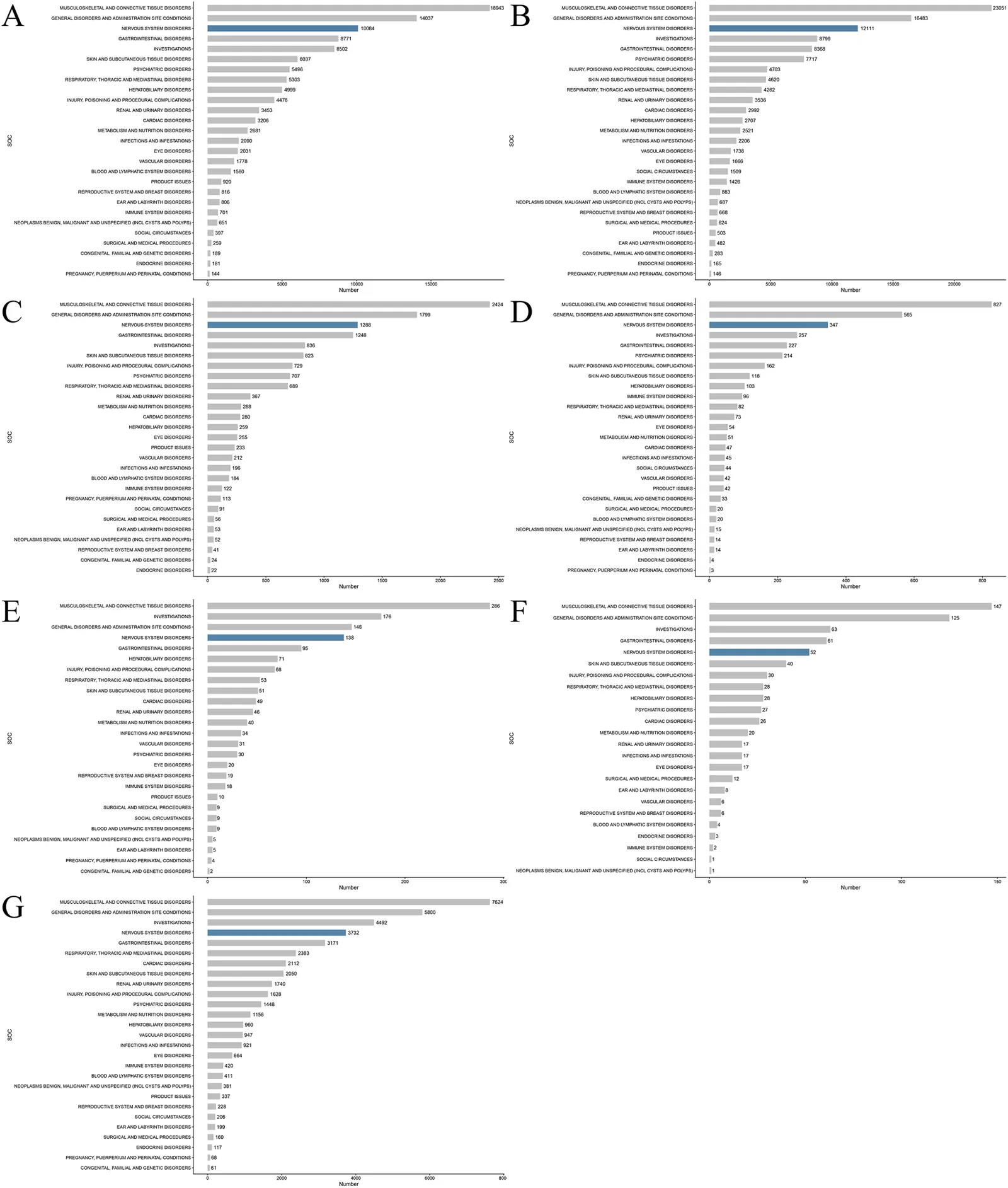
Signal detection of statins with positive significance at the system-organ classification (SOC) level. The results are presented using a bar chart. The height of the bar chart represents the quantity. Labels **(A-G)** correspond to Atorvastatin, Simvastatin, Pravastatin, Lovastatin, Fluvastatin, Pitavastatin and Rosuvastatin, respectively.

This study conducted a postmarketing pharmacovigilance analysis based on the FAERS database from the first quarter of 2005 to the first quarter of 2025, incorporating a total of 56,401 adverse event reports related to statins (Figure 1; Table 1). A total of 22,504,789 DEMO records were initially included, and 18,636,272 records were retained after deduplication. The top two drugs by number of reports were atorvastatin (n = 23,074) and simvastatin (n = 20,994). Among these, there were a total of 1,108 valid reports coded as “Amnesia” according to the MedDRA standard. Classification according to the System Organ Class (SOC) revealed that statin-related adverse events could affect 27 organ systems (Figure 2). MUSCULOSKELETAL AND CONNECTIVE TISSUE DISORDERS were the most common, while NERVOUS SYSTEM DISORDERS—comprising amnesia events—were also a major affected system category for statin adverse events.

**TABLE 1:** The formula to calculate ROR (95%CI),PRR (χ^2^),EBGM(EBGM05) and IC(IC025).

Name of algorithm	Formula	Positive signal criteria
ROR	ROR = ad/bc	Lower limit of 95%CI > 1, N ≥ 3
	95%CI = e ^ln(ROR)±1.96(1/a+1/b+1/c+1/d)^0.5^	
PRR	PRR=(a (c + d))/(c (a + b))	PRR ≥ 2, X^2^ ≥ 4, N ≥ 3
	*χ2* = [(ad-bc)^2^](a + b + c + d)/[(a + b) (c + d) (a + c) (b + d)]	
BCPNN	IC = log_2_a (a + b + c + d) (a + c) (a + b)	IC_025_ > 0
	IC_025_ = e ^ln(IC)−1.96(1/a+1/b+1/c+1/d)^0.5^	
MGPS	EBGM = a (a + b + c + d)/((a + c)/(a + b))	EBGM05 > 2, N > 0
	EBGM05 = e ^ln(EBGM)−1.64(1/a+1/b+1/c+1/d)^0.5^	

*ROR*, reporting odds ratio; *PRR*, proportional reporting ratio; *EBGM*, empirical Bayes geometric mean; *IC*, information component; *CI*, confidence interval; *95% CI*, two-sided for ROR; *χ2*, chi-squared; *EBGM05 and IC025*, lower one-sided for EBGM, and IC, respectively.

### 3.2 Signal mining

**FIGURE 3:**
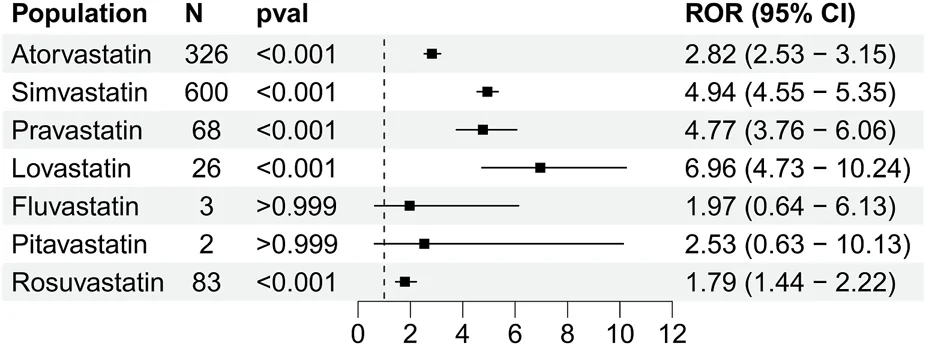
Forest plot showing the pharmacovigilance signals of statins at the overall level.

At the preferred term (PT) level, a disproportionality analysis was conducted on the association between seven statin drugs and amnesia; the results indicated significant heterogeneity in signal strength across different statin types (Figure 3; Table 2). Specifically, atorvastatin, simvastatin, pravastatin, and lovastatin all exhibited statistically significant positive warning signals; however, fluvastatin, pitavastatin, and rosuvastatin did not reach the positive threshold. Although rosuvastatin failed to meet the final composite threshold for positive signals defined in this study, it yielded a statistically significant positive result in the ROR analysis.

**TABLE 2:** Clinical distribution characteristics of adverse event reports associated with statins.

Characteristics (N,%)	Atorvastatin	Simvastatin	Pravastatin	Lovastatin	Fluvastatin	Pitavastatin	Rosuvastatin
Number of events	23,074	20,994	2,763	808	295	176	8,291
Sex,n%							
Female	9,455 (41.0%)	8,586 (40.9%)	1,207 (43.7%)	414 (51.2%)	122 (41.4%)	43 (24.4%)	3,472 (41.9%)
Male	10,758 (46.6%)	10,665 (50.8%)	1,125 (40.7%)	331 (41.0%)	140 (47.5%)	33 (18.8%)	3,865 (46.6%)
Unknown	2,861 (12.4%)	1743 (8.3%)	431 (15.6%)	63 (7.8%)	33 (11.2%)	100 (56.8%)	954 (11.5%)
Age,n%							
<18	71 (0.3%)	76 (0.4%)	17 (0.6%)	7 (0.9%)	1 (0.3%)	0 (0.0%)	40 (0.5%)
18–64.9	7,417 (32.1%)	7,004 (33.4%)	766 (27.7%)	242 (30.0%)	84 (28.5%)	26 (14.8%)	2,751 (33.2%)
65–85	9,736 (42.2%)	8,303 (39.5%)	980 (35.5%)	226 (28.0%)	111 (37.6%)	34 (19.3%)	3,368 (40.6%)
>85	884 (3.8%)	721 (3.4%)	82 (3.0%)	22 (2.7%)	4 (1.4%)	4 (2.3%)	201 (2.4%)
Unknow	4,966 (21.5%)	4,890 (23.3%)	918 (33.2%)	311 (38.5%)	95 (32.2%)	112 (63.6%)	1931 (23.3%)
Outcome							
CA	40 (0.2%)	44 (0.2%)	7 (0.3%)	2 (0.2%)	0 (0.0%)	0 (0.0%)	12 (0.1%)
DE	940 (4.1%)	658 (3.1%)	92 (3.3%)	15 (1.9%)	1 (0.3%)	13 (7.4%)	234 (2.8%)
DS	1,623 (7.0%)	1733 (8.3%)	138 (5.0%)	30 (3.7%)	8 (2.7%)	17 (9.7%)	482 (5.8%)
HO	6,380 (27.7%)	3,773 (18.0%)	409 (14.8%)	44 (5.4%)	37 (12.5%)	30 (17.0%)	1871 (22.6%)
LT	1,221 (5.3%)	773 (3.7%)	109 (3.9%)	12 (1.5%)	8 (2.7%)	9 (5.1%)	468 (5.6%)
OT	10,830 (46.9%)	5,773 (27.5%)	881 (31.9%)	106 (13.1%)	102 (34.6%)	71 (40.3%)	3,926 (47.4%)
RI	45 (0.2%)	17 (0.1%)	4 (0.1%)	2 (0.2%)	0 (0.0%)	0 (0.0%)	35 (0.4%)
Unknown	1995 (8.6%)	8,223 (39.2%)	1,123 (40.6%)	597 (73.9%)	139 (47.1%)	36 (20.5%)	1,263 (15.2%)

Abbreviations: CA: congenital anomaly, DE: death, DS: disability, HO: Hospitalization-Initial or Prolonged, LT: Life-Threatening, OT: other serious important medical event, RI: required intervention to prevent permanent.

**FIGURE 4:**
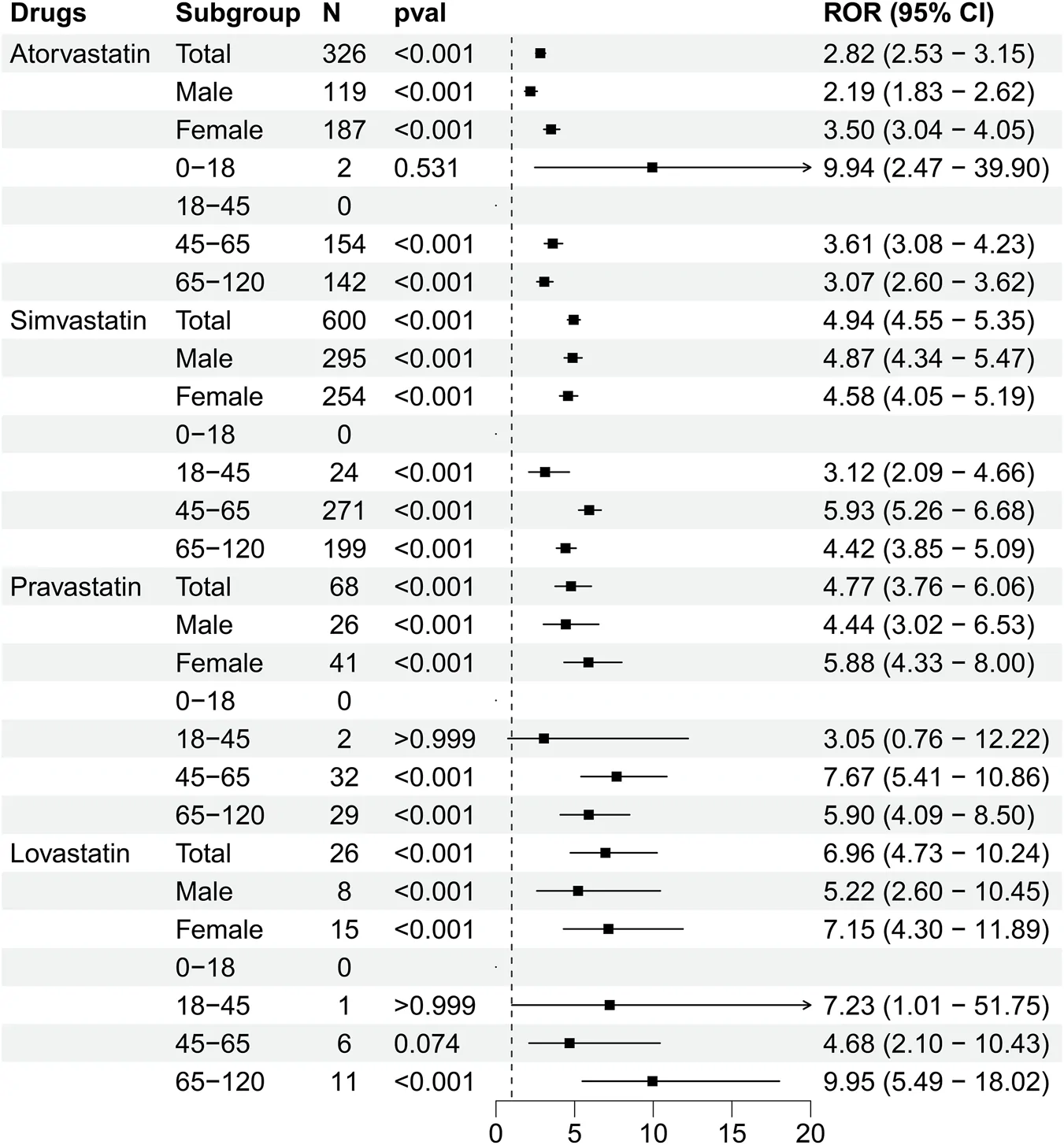
Forest plot showing the subgroup-level pharmacovigilance signals of 4 statin drugs that presented positive signals at the overall level.

Furthermore, subgroup analysis by sex revealed that female participants reported a higher number of amnesia cases and exhibited a stronger signal overall compared to males; this difference was particularly pronounced in the atorvastatin group (Figure 4). The signal of amnesia associated with atorvastatin was significantly stronger in women (n = 187, ROR = 3.50, 95% CI: 3.04–4.05, P < 0.001) than in men (n = 119, ROR = 2.19, 95% CI: 1.83–2.62, P < 0.001).

Age-stratified analysis indicated that positive signals were observed in most statin subgroups among individuals aged 45–65 and ≥65 years. Notably, for lovastatin in the 45–65 age group, the result yielded a P value of 0.074 after Bonferroni correction and thus did not reach statistical significance. Whereas all signals were negative in the 0–18 age group. In the signal analysis of this age subgroup, atorvastatin yielded a positive signal only via the EBGM algorithm due to a limited case count of 2, which failed to meet our criteria for a valid positive signal. By contrast, no amnesia-related adverse events were reported for other statins within this subgroup. It is noteworthy that rosuvastatin presented a positive signal exclusively in the ROR analysis at the total level. When stratified by age into the 45–65 years subgroup, positive pharmacovigilance signals were identified by three disproportionality approaches including ROR, whereas the EBGM algorithm failed to detect a positive association. Notably, when focusing on the 18–45-year-old subgroup, a positive signal was detected only in the simvastatin group (n = 24, ROR = 3.12, 95% CI: 2.09–4.66, P < 0.001). Importantly, no amnesia-related reports of atorvastatin were retrieved for this 18–45 age subgroup. This phenomenon is likely associated with relatively low prescription volume and limited drug exposure among young and middle-aged populations. Additionally, fluvastatin, pitavastatin, and rosuvastatin, which showed negative signals in the overall population, remained consistently non-significant across all subgroup analyses.

### 3.3 Analysis of onset time

**FIGURE 5:**
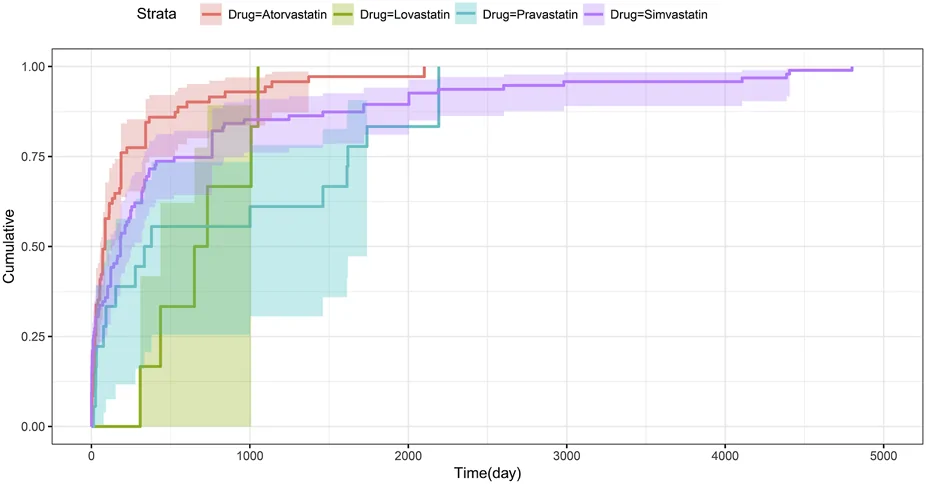
Cumulative distribution function of 4 statin drugs by time-to-onset.

The Weibull distribution model was used to fit the temporal patterns of adverse events, and the results showed that adverse events associated with different statins exhibited distinct temporal distribution patterns (Figure 5; Table 3). Atorvastatin and lovastatin exhibited early-onset failure characteristics, with adverse events concentrated in the early stages of treatment and a decreasing risk trend as exposure time increased; the corresponding scale parameters indicated typical onset times of 5.6 months for atorvastatin and 10.0 months for lovastatin. Simvastatin and pravastatin, however, exhibited random failure characteristics, with a more dispersed distribution of event occurrence times and no obvious tendency toward temporal clustering, with typical onset times of 2.1 years for simvastatin and 2.0 years for pravastatin.

**TABLE 3:** Baseline denominators across all subgroups.

Drugs	Subgroup	a	b	c	d
Atorvastatin	Total	326	108,185	83,083	77,846,098
	Male	119	49,972	28,510	26,196,911
	Female	187	47,430	49,577	44,072,429
	0–18	2	446	1,411	3,126,659
	18–45	0	​	​	​
	45–65	154	37,903	22,045	19,576,787
	65–120	142	49,696	16,210	17,392,039
Simvastatin	Total	600	114,256	82,809	77,840,027
	Male	295	55,957	28,334	26,190,926
	Female	254	49,318	49,510	44,070,541
	0–18	0	​	​	​
	18–45	24	6,509	14,042	11,889,108
	45–65	271	40,819	21,928	19,573,871
	65–120	199	48,434	16,153	17,393,301
Pravastatin	Total	68	13,323	83,341	77,940,960
	Male	26	5,370	28,603	26,241,513
	Female	41	6,183	49,723	44,113,676
	0–18	0	​	​	​
	18–45	2	555	14,064	11,895,062
	45–65	32	3,693	22,167	19,610,997
	65–120	29	5,251	16,323	17,436,484
Lovastatin	Total	26	3,493	83,383	77,950,790
	Male	8	1,406	28,621	26,245,477
	Female	15	1860	49,749	44,117,999
	0–18	0	​	​	​
	18–45	1	117	14,065	11,895,500
	45–65	6	1,134	22,193	19,613,556
	65–120	11	1,180	16,341	17,440,555
Fluvastatin	Total	3	1,421	83,406	77,952,862
	Male	2	652	28,627	26,246,231
	Female	1	649	49,763	44,119,210
	0–18	0	​	​	​
	18–45	0	​	​	​
	45–65	0	​	​	​
	65–120	3	604	16,349	17,441,131
Pitavastatin	Total	2	739	83,407	77,953,544
	Male	0	​	​	​
	Female	0	​	​	​
	0–18	0	​	​	​
	18–45	0	​	​	​
	45–65	0	​	​	​
	65–120	0	​	​	​
Rosuvastatin	Total	83	43,333	83,326	77,910,950
	Male	39	19,312	28,590	26,227,571
	Female	32	19,556	49,732	44,100,303
	0–18	0	​	​	​
	18–45	3	2086	14,063	11,893,531
	45–65	41	15,556	22,158	19,599,134
	65–120	23	18,195	16,329	17,423,540

Cell a represents the number of reported cases exposed to the target MS treatment drugs who developed amnesia; cell b represents the number of reported cases exposed to the target MS treatment drugs who did not develop amnesia; cell c represents the number of reported cases exposed to other drugs who developed amnesia; and cell d represents the number of reported cases exposed to other drugs who did not develop amnesia.

## 4 Discussion

As first-line medications for the clinical management of dyslipidemia, statins have been widely used in the long-term treatment of hypercholesterolemia. Although their efficacy is well-established, a series of clinical case reports have suggested that some patients may experience amnesia after taking statins, indicating a potential association between these drugs and amnesia (Healy et al., 2009; Li et al., 2018). Nevertheless, some randomized controlled trials have yielded inconsistent results. Specifically the ASPREE, HOPE-3, and JUPITER studies, have demonstrated that long-term statin therapy is not significantly associated with increased risk of long-term cognitive decline (Ratchford and Martin, 2017; Bosch et al., 2019; Hoshi et al., 2024; Brodtmann et al., 2025). These contradictory findings between clinical adverse reports and trials evidence indicate that the cognitive safety profile of statins remains controversial and not fully clarified. Additionally, temporal risk characteristics, and clinical implications of statin-related amnesia remain poorly systematically elucidated. Given the extensive prescription coverage and prolonged treatment cycle of statins in clinical practice, as well as the fact that amnesia can severely impair patients’ daily cognitive function and quality of life, a systematic post-marketing evaluation focusing on the reporting signals of statin-associated amnesia is of substantial clinical value and practical necessity. Accordingly, this study adopted a pharmacovigilance research framework and performed disproportionality analysis based on real-world FAERS adverse event data to systematically explore the reporting association features between commonly used statins and amnesia, aiming to supplement current clinical evidence and provide targeted safety references for long-term statin medication management.

When mining signals in the overall population, our results indicate that atorvastatin, simvastatin, pravastatin, and lovastatin all exhibited statistically significant positive warning signals (Table 4). In contrast, fluvastatin, pitavastatin, and rosuvastatin did not meet the threshold for a positive determination. There is significant heterogeneity in the signals linking the aforementioned statins to amnesia, which can be reasonably explained by factors such as the drugs’ physicochemical properties, levels of exposure in the central nervous system, and clinical application characteristics, as well as universal confounding factors including advanced age and pre-existing cardiovascular and cerebrovascular comorbidities shared by the major user population of these statins.

**TABLE 4:** Subgroup analysis of statins based on age and gender.

Drugs	Subgroup	N	ROR (95%Cl)	PRR (χ^2^)	EBGM(EBGM05)	IC(IC025)	Bonferron_P_value
Atorvastatin	Total	326	2.82 (2.53–3.15)	2.82 (381.25)	2.81 (2.52)	1.49 (1.32)	5.27372828974617e-81
	Male	119	2.19 (1.83–2.62)	2.19 (76.27)	2.18 (1.82)	1.12 (0.85)	1.05631001286597e-14
	Female	187	3.5 (3.04–4.05)	3.5 (332.2)	3.49 (3.02)	1.8 (1.57)	2.40779387387064e-70
	0–18	2	9.94 (2.47–39.9)	9.9 (15.98)	9.88 (2.46)	3.31 (-0.35)	0.531160232564761
	18–45	0	​	​	​	​	​
	45–65	154	3.61 (3.08–4.23)	3.6 (287.15)	3.58 (3.05)	1.84 (1.58)	1.53277562024386e-60
	65–120	142	3.07 (2.6–3.62)	3.06 (195.38)	3.04 (2.58)	1.61 (1.34)	1.27855437396149e-40
Simvastatin	Total	600	4.94 (4.55–5.35)	4.92 (1860)	4.89 (4.51)	2.29 (2.16)	<0.001
	Male	295	4.87 (4.34–5.47)	4.85 (894.05)	4.81 (4.29)	2.27 (2.08)	2.93341280087154e-192
	Female	254	4.58 (4.05–5.19)	4.57 (704.59)	4.55 (4.02)	2.19 (1.98)	3.77774205683073e-151
	0–18	0	​	​	​	​	​
	18–45	24	3.12 (2.09–4.66)	3.11 (34.43)	3.11 (2.08)	1.64 (0.94)	9.02179899693171e-06
	45–65	271	5.93 (5.26–6.68)	5.89 (1,088.99)	5.83 (5.17)	2.54 (2.34)	1.75091562500545e-234
	65–120	199	4.42 (3.85–5.09)	4.41 (518.84)	4.37 (3.8)	2.13 (1.9)	8.34530080369813e-111
Pravastatin	Total	68	4.77 (3.76–6.06)	4.75 (201.63)	4.75 (3.74)	2.25 (1.82)	7.45446927307251e-42
	Male	26	4.44 (3.02–6.53)	4.43 (68.95)	4.42 (3.01)	2.14 (1.42)	4.44482392849857e-13
	Female	41	5.88 (4.33–8)	5.85 (164.94)	5.85 (4.3)	2.55 (1.94)	8.76446914964898e-34
	0–18	0	​	​	​	​	​
	18–45	2	3.05 (0.76–12.22)	3.04 (2.74)	3.04 (0.76)	1.6 (-0.82)	>0.999
	45–65	32	7.67 (5.41–10.86)	7.61 (183.63)	7.6 (5.36)	2.93 (2.16)	1.28547034909433e-37
	65–120	29	5.9 (4.09–8.5)	5.87 (117.14)	5.86 (4.07)	2.55 (1.81)	2.31638712657275e-23
Lovastatin	Total	26	6.96 (4.73–10.24)	6.91 (131.64)	6.91 (4.7)	2.79 (1.95)	2.38221173798564e-26
	Male	8	5.22 (2.6–10.45)	5.19 (27.11)	5.19 (2.59)	2.38 (0.86)	0.000639498020112449
	Female	15	7.15 (4.3–11.89)	7.1 (78.71)	7.1 (4.27)	2.83 (1.64)	6.45314132317017e-15
	0–18	0	​	​	​	​	​
	18–45	1	7.23 (1.01–51.75)	7.18 (5.32)	7.18 (1)	2.84 (-1.25)	>0.999
	45–65	6	4.68 (2.1–10.43)	4.66 (17.24)	4.66 (2.09)	2.22 (0.52)	0.0742832655570561
	65–120	11	9.95 (5.49–18.02)	9.87 (87.67)	9.86 (5.44)	3.3 (1.67)	2.21919041563287e-16
Fluvastatin	Total	3	1.97 (0.64–6.13)	1.97 (1.44)	1.97 (0.63)	0.98 (-0.78)	>0.999
	Male	2	2.81 (0.7–11.27)	2.81 (2.33)	2.81 (0.7)	1.49 (-0.86)	>0.999
	Female	1	1.37 (0.19–9.71)	1.37 (0.1)	1.37 (0.19)	0.45 (-1.84)	>0.999
	0–18	0	​	​	​	​	​
	18–45	0	​	​	​	​	​
	45–65	0	​	​	​	​	​
	65–120	3	5.3 (1.7–16.48)	5.28 (10.41)	5.28 (1.7)	2.4 (-0.1)	>0.999
Pitavastatin	Total	2	2.53 (0.63–10.13)	2.53 (1.84)	2.53 (0.63)	1.34 (-0.93)	>0.999
	Male	0	​	​	​	​	​
	Female	0	​	​	​	​	​
	0–18	0	​	​	​	​	​
	18–45	0	​	​	​	​	​
	45–65	0	​	​	​	​	​
	65–120	0	​	​	​	​	​
Rosuvastatin	Total	83	1.79 (1.44–2.22)	1.79 (28.91)	1.79 (1.44)	0.84 (0.51)	0.000268815583524878
	Male	39	1.85 (1.35–2.54)	1.85 (15.25)	1.85 (1.35)	0.89 (0.4)	0.230451015837018
	Female	32	1.45 (1.03–2.05)	1.45 (4.48)	1.45 (1.02)	0.54 (0.01)	>0.999
	0–18	0	​	​	​	​	​
	18–45	3	1.22 (0.39–3.77)	1.22 (0.12)	1.22 (0.39)	0.28 (-1.24)	>0.999
	45–65	41	2.33 (1.72–3.17)	2.33 (31.03)	2.33 (1.71)	1.22 (0.73)	6.96777370374637e-05
	65–120	23	1.35 (0.9–2.03)	1.35 (2.07)	1.35 (0.9)	0.43 (-0.18)	>0.999

Abbreviations: ROR: report odds ratio, PRR: proportional reporting ratio, IC: Information Component, EBGM: Empirical Bayesian Geometric Mean and P value: Adjusted P value.

Specifically, atorvastatin, simvastatin, and lovastatin are all lipid-soluble statins with strong blood-brain barrier permeability (Fujimoto et al., 2021; Shen et al., 2025). They can enter the central nervous system and inhibit cholesterol synthesis in the brain, thereby affecting neuronal membrane stability, synaptic transmission, and mitochondrial energy metabolism, which may increase the risk of adverse events related to memory impairment and amnesia (Fong, 2014).

Notably, these three agents are predominantly prescribed to elderly patients and individuals with multiple chronic illnesses; such population characteristics may also introduce confounding effects related to age and comorbidities when interpreting the detected positive signals. Additionally, although pravastatin is a hydrophilic statin, due to its early market release, widespread clinical use, and particularly common use in the elderly and high-risk populations with concomitant cardiovascular and cerebrovascular diseases, positive warning signals may still be detected due to confounding factors such as underlying diseases and age, which is consistent with the confounding patterns observed for atorvastatin, simvastatin and lovastatin (Adams et al., 2023).

Besides, as illustrated in the methodological section, the ROR method possesses superior performance in minimizing analytical bias. In terms of pharmacovigilance signal interpretation, rosuvastatin presented a statistically significant positive signal in ROR disproportionality analysis, yet failed to yield positive outcomes in the other three algorithms. Such inconsistent findings differ distinctly from fluvastatin and pitavastatin, both of which consistently showed negative signals across all analyses including the ROR model. This discrepancy indicates that the cognitive safety profile of rosuvastatin warrants further in-depth subgroup analysis.

The number of reported cases of amnesia and the associated signal were slightly higher in female subjects than in male subjects overall. This difference was particularly pronounced in the atorvastatin group, which had a higher number of reports. This gender difference may be related to various factors, including pharmacokinetic characteristics, sex hormone levels, central nervous system sensitivity, and clinical reporting bias. Women exhibit inherent physiological differences in body weight, body fat percentage, and hepatic drug-metabolizing enzyme activity. Consequently, they have a lower clearance rate for statins such as atorvastatin, which are metabolized via the CYP3A4 pathway. This may contribute to increased systemic drug exposure and tentatively raise the possibility of drug accumulation within the central nervous system (Willrich et al., 2009; Yoon et al., 2021). Concurrently, estrogen levels in women fluctuate significantly with age. As this hormone plays a crucial regulatory role in hippocampal neuronal function, synaptic plasticity, and cholinergic neurotransmission, declining estrogen levels can weaken the protective effects on the central nervous system (Bean et al., 2014; Brann et al., 2021; Sochocka et al., 2023). These physiological differences may tentatively suggest a potential higher susceptibility of females to statin-related neurotoxic responses including disrupted cerebral cholesterol metabolism (Palmisano et al., 2017). Furthermore, women may more sensitive to subjective perceptions of cognitive dysfunction, and their willingness and rate of reporting adverse events are typically higher than those of men, objectively leading to gender imbalance in reported data (Brabete et al., 2022).

Age-stratified analysis revealed positive signals in the statin subgroup of individuals aged ≥45 years, whereas the 0–18-year-old group showed negative results. Likewise, we observed a comparable pattern when analyzing rosuvastatin within the 45–65 age subgroup. While the relevant indicators failed to reach the standard for a positive event signal formulated in our research, this subgroup demonstrated a markedly higher signal value relative to younger age groups. Specifically, three of the four signal detection algorithms yielded positive results here, and the statistical metrics reached significant levels after Bonferroni correction. Such a borderline yet elevated signal does not constitute conclusive evidence of definite adverse risks, but it may reflects a potential growing tendency of cognitive adverse events among middle-aged patients exposed to rosuvastatin. Combined with the inconsistent signal performance of rosuvastatin across different algorithms, this finding reinforces the necessity of further investigating its cognitive safety in middle-aged populations. The characteristics of the association signals observed in the age-stratified analysis may be directly related to the indications for use, drug exposure levels, physiological functions, and central nervous system status in each age group. The 0–18 age group is not the conventional target population for statins; clinical drug exposure rates and cumulative duration of use are extremely low, and sensitivity to the central nervous system effects of statins may be lower, which is why no positive signals related to amnesia were detected.

In contrast, individuals aged 45–65 and ≥65 years are at high risk for atherosclerotic cardiovascular disease and constitute the core target population for long-term statin therapy, with significantly higher levels of drug exposure and duration of use. Additionally, with advancing age, the activity of hepatic metabolic enzymes decreases and the permeability of the blood-brain barrier increases, which can lead to the accumulation of statins in the central nervous system following long-term use (Klotz, 2009; Montagne et al., 2015). Furthermore, this population often has comorbidities such as hypertension, diabetes, and cerebral small-vessel disease, along with reduced cognitive reserve and neural compensatory capacity. These multiple factors collectively drive an increased detection rate of amnesia-related signals, resulting in a positive signal observed in most statin subgroups (Fan et al., 2024).

It is worth noting that individuals aged 18–45 are in the prime of their cognitive reserve; during this age range, the central nervous system is fully developed, and neural compensatory capacity is well-established. At this stage, the incidence of endogenous amnesia caused by age-related cognitive decline, neurodegenerative diseases, and cerebral small vessel disease is low. Conducting analysis on this age subgroup minimizes the interference of confounding factors—such as underlying comorbidities—in the attribution of adverse events. Therefore, positive signals detected in this subgroup may more accurately reflect a potential association relationship between the drug and amnesia.

Our results indicate that, within the 18–45-year-old subgroup, only the simvastatin group exhibited a positive warning signal for amnesia; this finding demonstrates clear drug-specificity and potential clinical risk implications. The underlying mechanism can be systematically elucidated by integrating the physiological characteristics of this age group, the pharmacological properties of simvastatin, and structural differences among various statins. From a pharmacological perspective, simvastatin is a highly lipophilic statin with strong passive penetration capacity through the blood-brain barrier. It can effectively enter the central nervous system and inhibit HMG-CoA reductase activity in the brain, which may subsequently interfere with neuronal cholesterol synthesis, disrupt synaptic membrane stability, and affect mitochondrial energy metabolism, ultimately directly triggering memory impairment (Fong, 2014). Additionally, simvastatin is primarily metabolized by hepatic CYP3A4; even in young and middle-aged individuals with robust metabolic function, it can achieve stable and effective central nervous system drug exposure.

In contrast, no positive signals were detected for the other six statins in this subgroup, a finding that may be attributed to the following factors. First, some statins are highly hydrophilic, resulting in extremely low blood-brain barrier permeability and thus an inability to achieve effective drug exposure in the central nervous system. Additionally, the clinical indications for some statins are limited, leading to a small exposure base in the young and middle-aged population, making it difficult to detect statistically significant risk signals. Furthermore, although some statins are lipophilic, the duration of use and cumulative dose in young and middle-aged adults were insufficient to reach the threshold for detectable central nervous system toxicity. Additionally, drug interactions may be one of the key reasons for the positive association observed with simvastatin in this subgroup. Simvastatin is primarily metabolized by CYP3A4 and is frequently co-administered with CYP3A4 inhibitors such as itraconazole and ritonavir in clinical practice. Such combined medication may raise simvastatin plasma concentration and systemic exposure, which might facilitate its accumulation in the central nervous system and potentially imply a tentative correlation with elevated amnesia-related adverse event signals (Hirota et al., 2020).

The aforementioned study results indicate a positive pharmacovigilance association between simvastatin and amnesia, and a clear positive signal persists even after excluding the confounding effects of age-related cognitive decline in the elderly population. This suggests a potentially closer relationship between simvastatin and adverse events related to memory impairment. Nevertheless, it should be emphasized that spontaneous reporting databases cannot accomplish refined adjustment for individual-level influencing factors including patient comorbidities and baseline cognitive status. Hence, the observed disproportionality signals are merely statistical reporting associations rather than definitive quantitative clinical risk evidence. Relevant findings are only exploratory hypothesis-generating clues, which still require further well-designed pharmacoepidemiological studies for validation.

Atorvastatin and lovastatin exhibited early failure patterns, with adverse events concentrated in the early stages of treatment; the potential risk showed a decreasing trend as exposure time increased. In contrast, simvastatin and pravastatin exhibited a random failure pattern, with event occurrence distributed more widely and no clear tendency toward temporal clustering. The sample sizes for Weibull distribution fitting of each statin are presented in Table 5. Notably, simvastatin and pravastatin had relatively smaller fitting sample sizes, resulting in wider confidence intervals and lower statistical precision for their temporal parameter estimates. Despite this analytical limitation, these results still validly reflect the distinct temporal characteristics of adverse events associated with different statins in real-world spontaneous reports, which aligns with the exploratory nature of this pharmacovigilance study.

**TABLE 5:** Weibull parameter test for amnesia -related adverse events associated with statins.

Drugs	Shape parameter (95% CI)	Scale parameter (95% CI)	N	Type
Atorvastatin	0.649 (0.536–0.761)	167.617 (103.978–231.256)	71	Early failure
Simvastatin	2.872 (0.987–4.757)	786.048 (555.386–1,016.709)	6	Random failure
Pravastatin	0.724 (0.447–1.002)	716.276 (235.092–1,197.459)	18	Random failure
Lovastatin	0.499 (0.419–0.579)	301.117 (173.305–428.928)	95	Early failure

*CI*, confidence interval; *95% CI*, two-sided for Shape parameter and Scale parameter.

Clinically, these temporal characteristics further highlight the necessity of distinguishing transient drug-related adverse reactions from progressive neurodegenerative disorders. Specifically, transient adverse drug reactions refer to symptomatic impairments that emerge after drug administration and are reversible upon treatment discontinuation (Hirschfeld et al., 2007; Ratchford and Martin, 2017; Ropper, 2023). In contrast, progressive neurodegenerative diseases are predominantly characterized by irreversible neurological functional decline, whose progression is closely correlated with chronic disease course (Hou et al., 2019; Serio et al., 2025). Notably, the early-onset and time-dependent attenuating pattern of cognitive events observed for certain statins is consistent with the features of acute, drug-induced reversible cognitive symptoms rather than chronic degenerative damage. Nevertheless, the occurrence of such transient cognitive symptoms during long-term statin therapy may compromise patients’ daily quality of life and reduce long-term medication adherence. More importantly, the identified adverse event reporting signals provide critical pharmacovigilance evidence for early identification of high-risk populations, scheduled cognitive function monitoring during prolonged treatment, and individualized medication decision-making, which is conducive to improving and safeguarding the long-term medication safety of patients receiving statin therapy.

It's worth noting that the Weber effect should be taken into consideration (Hoffman et al., 2014). This phenomenon refers to the tendency that newly marketed drugs tend to receive more adverse event reports in the early post-approval period. Since rosuvastatin (launched in 2003) and pitavastatin (launched in 2009) obtained marketing approval later than other included statins, the Weber effect may interfere with the comparison of their adverse event reporting rates and signal results across different agents. Furthermore, the present study lies in the adoption of all other drugs recorded in the FAERS database as the general comparator group. This broad comparator cohort contains heterogeneous drug categories, diverse patient populations, and varying reporting patterns across different therapeutic agents, which may introduce systematic reporting bias. Such study design limitations may affect the magnitude of disproportionality signals. It is also important that given the established cardiovascular benefits of statins, clinicians should avoid overinterpreting the observed cognitive adverse event signals. Statins are first-line drugs for LDL-C control and cardiovascular prevention, and unnecessary treatment cessation caused by suspected adverse events will raise cardiovascular risks. Considering the complexity of statin intolerance and adverse event attribution, our results do not contradict standard statin prescription for indicated patients. Instead, we recommend enhanced clinical surveillance for those experiencing cognitive complaints during treatment, alongside rigorous assessment, patient education, drug rechallenge and personalized lipid-lowering regimens.

The management of statin intolerance requires a patient-centered approach to formulate individualized therapeutic plans (Bosco et al., 2023). For patients with mild intolerance, switching statin varieties, reducing dosage or adopting intermittent administration can be prioritized. For those who cannot tolerate any statin preparation at any dose, or fail to achieve target LDL-C levels even on the maximally tolerated statin dose, non-statin lipid-lowering agents are viable alternatives to maintain cardiovascular protection (Bosco et al., 2023). Currently available options include ezetimibe, PCSK9 inhibitors such as alirocumab, evolocumab and inclisiran, as well as bempedoic acid and nutraceuticals (Bosco et al., 2023). Accumulated evidence has demonstrated that combining PCSK9 inhibitors with high-intensity statins and ezetimibe can effectively reduce LDL-C and lipoprotein(a) levels and improve vascular conditions in patients with familial hypercholesterolemia (Bosco et al., 2025). Collectively, the findings of the present pharmacovigilance study suggest optimizing lipid-lowering strategies rather than discontinuing treatment blindly, so as to balance medication safety and long-term cardiovascular prevention.

This study, which relies on the FAERS real-world adverse event database to conduct a pharmacovigilance analysis of the potential association between statins and amnesia, offers several research advantages. First, by incorporating real-world data spanning a long time period and involving a large sample size, this study objectively reflects the characteristics of amnesia-related adverse events during the widespread clinical use of statins, ensuring that the results possess good external validity and representativeness. Second, this study employs a multidimensional analytical strategy, systematically examining descriptive statistics, signal detection, stratification by gender and age, and temporal distribution patterns. By combining these analyses with the Weibull distribution model to quantify event occurrence patterns, the study provides a relatively reliable real-world basis for the neurocognitive safety assessment of statins, offering valuable reference for optimizing clinical drug selection and potential risk management.

At the same time, this study has several limitations. As a retrospective study based on a spontaneous reporting system, it can only suggest a statistical association between the drug and adverse events, but cannot establish a definitive causal relationship. Furthermore, the FAERS database itself has inherent shortcomings, such as underreporting, reporting bias, and the absence of certain key information. Notably, among the drugs examined in this study, pitavastatin exhibited a higher rate of missing demographic data, which may introduce relatively greater bias. Confounding factors are difficult to fully control and may impair the accuracy of signal detection. Specifically, spontaneous adverse event reporting databases cannot achieve refined adjustment for individual-level factors including patient comorbidities, baseline cognitive risk and disease severity. Similarly, although rosuvastatin did not strictly meet the threshold for a positive adverse event signal defined in this study, its signal has reached a near-threshold level. Besides, fluvastatin and pitavastatin had relatively low adverse event reporting volumes in the FAERS dataset. In this context, the non-significant signal results detected for these three statins merely indicate a lack of identifiable signals in the current database rather than a complete absence of potential amnesia-related adverse risks. Furthermore, using the general FAERS population as the reference group also limits the clinical interpretation of inter-statin signal discrepancies, since such differences are additionally affected by prescribing patterns, population composition and time on market. Future research should include prospective cohort studies, precision epidemiological investigations, and mechanistic experiments to further clarify the true risk and underlying mechanisms of statin-associated cognitive impairment, thereby providing a higher level of evidence-based support for the safe and rational use of medications in clinical practice.

## 5 Conclusion

This study identified positive pharmacovigilance signals linking amnesia to atorvastatin, simvastatin, pravastatin and lovastatin, whereas fluvastatin, pitavastatin and rosuvastatin failed to meet the signal threshold. Stronger signals were observed among female and middle-aged populations. Distinct temporal patterns of adverse events were also found across agents, suggesting targeted safety monitoring during different treatment phases. Given inherent limitations and confounding biases of spontaneous reporting databases, definitive causal inference cannot be drawn. These findings serve as exploratory clues for subsequent safety research. Well-designed prospective cohort studies and pharmacoepidemiological analyses are required to adjust for confounders and quantify real-world risks. Further mechanistic experiments are also needed to clarify the molecular and neural mechanisms underlying statin-related cognitive impairment.

## Data Availability

The original contributions presented in the study are included in the article/Supplementary Material, further inquiries can be directed to the corresponding author.
