# Supplementary material for: Statins and reporting signals for amnesia: a pharmacovigilance analysis based on adverse event reporting database
Source: Front Pharmacol. 2026 Aug 11;17:1858243. doi: 10.3389/fphar.2026.1858243 (PMC13503598; doi:10.3389/fphar.2026.1858243)
Supplement: Supplementary file 1 [file Table1.docx]

**Table S1** Search terms (generic and brand names) used for drug retrieval.

| **Atorvastatin** | **Simvastatin** | **Pravastatin** | **Lovastatin** | **Fluvastatin** | **Pitavastatin** | **Rosuvastatin** |
| --- | --- | --- | --- | --- | --- | --- |
| ATORVASTATIN CALCIUM | SIMVASTATIN | PRAVASTATIN | LOVASTATIN | FLUVASTATIN SODIUM | PITAVASTATIN CALCIUM | ROSUVASTATIN CALCIUM |
| ATORVASTATIN | EZETIMIBE SIMVASTATIN | PRAVASTATIN SODIUM | LOVASTATIN TABLETS USP | FLUVASTATIN | PITAVASTATIN | ROSUVASTATIN |
| ATORVASTATIN ATORVASTATIN CALCIUM | SIMVASTATIN UNKNOWN | PRAVASTATIN PRAVASTATIN SODIUM |  |  | PITAVASTATIN CALCIUM | ROSUVASTATIN ROSUVASTATIN CALCIUM |
| ATORVASTATIN CALCIUM EZETIMIBE | SIMVASTATIN SIMVASTATIN | PRAVASTATINE SODIQUE |  |  | EZETIMIBE PITAVASTATIN CALCIUM | APO ROSUVASTATIN |
| ATORVASTATIN CALCIUM TABLETS | SIMVASTATIN TABLETS USP | PRAVASTATIN TABLET |  |  |  | ROSUVASTATIN FILM COATED TABLET |
| TAHOR ATORVASTATIN | SIMVASTATIN TABLETS | PRAVASTATIN SODIUM UNKNOWN |  |  |  | ROSUVASTATIN CALCIUM TABLETS |
| ATORVASTATIN ATORVASTATIN | EZETIMIBE AND SIMVASTATIN | PRAVASTATIN ACCORD |  |  |  | ROSUVASTATIN SANDOZ |
| SORTIS ATORVASTATIN | SIMVASTATIN FILM COATED TABLET |  |  |  |  | TEVA ROSUVASTATIN |
| ATORVASTATIN PFIZER | NIACIN SIMVASTATIN |  |  |  |  | ROSUVASTATIN TABLETS |
| ATORVASTATINA 7400A | SIMVASTATIN ACCORD |  |  |  |  | EZETIMIBE ROSUVASTATIN |
| ATORVASTATIN MYLAN | SIMVASTATIN WATSON LABORATORIES |  |  |  |  | ROSUVASTATIN ACCORD |
| APO ATORVASTATIN | SIMVASTATIN SANDOZ |  |  |  |  |  |
| ATORVASTATIN EZETIMIBE | SIMVASTATIN NGX |  |  |  |  |  |
| CITALOR ATORVASTATIN | SIMVASTATIN ACTAVIS |  |  |  |  |  |
| ATORVASTATIN SANDOZ | BLINDED EZETIMIBE SIMVASTATIN |  |  |  |  |  |
| ATORVASTATINA DOC GENERICI COMPRESSE RIVESTITE CON FILM | NIACIN ER SIMVASTATIN |  |  |  |  |  |
| ATORVASTATIN UNKNOWN | SIMVASTATIN TEVA |  |  |  |  |  |
| ATORVASTATIN TEVA |  |  |  |  |  |  |
| ATORVASTATIN ACCORD |  |  |  |  |  |  |
| ATORVASTATIN CALCIUM TRIHYDRATE |  |  |  |  |  |  |
| ATORVASTATIN ORION |  |  |  |  |  |  |
| ATORVASTATIN HEXAL |  |  |  |  |  |  |
| ZARATOR ATORVASTATIN |  |  |  |  |  |  |
| LIPITOR ATORVASTATIN |  |  |  |  |  |  |
